# The Effectiveness and Safety of Huangqi Xixin Decoction for Cough Variant Asthma: A Systematic Review and Meta-Analysis

**DOI:** 10.1155/2022/9492100

**Published:** 2022-09-20

**Authors:** Cong Wang, Qingqing Xia, Beina Hu, Weilong Jiang, Huizhe Zhang

**Affiliations:** ^1^Department of Pulmonary and Critical Care Medicine, Jiangyin Hospital of Traditional Chinese Medicine, Jiangyin Hospital Affiliated to Nanjing University of Chinese Medicine, Jiangyin, Jiangsu 214400, China; ^2^Department of Clinical Laboratory, Jiangyin Hospital of Traditional Chinese Medicine, Jiangyin Hospital Affiliated to Nanjing University of Chinese Medicine, Jiangyin, Jiangsu 214400, China; ^3^Department of Respiratory Medicine, Yancheng Hospital of Traditional Chinese Medicine, Yancheng Hospital Affiliated to Nanjing University of Chinese Medicine, Yancheng, Jiangsu 224005, China

## Abstract

**Objective:**

A comprehensive and systematic review is needed to evaluate the safety and effectiveness of Huangqi Xixin decoction (HQXXD) for cough variant asthma (CVA). In this systematic review, we comprehensively interrogate the safety and effectiveness of HQXXD for CVA.

**Methods:**

An overall search for studies in main English and Chinese electronic databases from their inception to June 30, 2022, was performed. Randomized controlled trials (RCTs) involving HQXXD for CVA were included. According to Cochrane Reviewer's Handbook, the risk of bias related to the included studies was evaluated. A meta-analysis using RevMan 5.4 software from the Cochrane Collaboration was used to integrate the outcomes of the included RCTs.

**Results:**

A systematic review and meta-analysis were conducted using the seven eligible RCTs that had been retrieved. The included RCT-related risk of bias was evaluated. According to the findings of the meta-analysis, the HQXXD group had significantly higher total effective rates of clinical efficacy and airway responsiveness, and a significantly lower recurrence rate in comparison with the conventional Western medicine treatment group.

**Conclusion:**

In the treatment of CVA patients, HQXXD is safe and effective, which benefits clinical efficacy and airway responsiveness, reduces the recurrence rate, and has no adverse effects.

## 1. Introduction

Cough variant asthma (CVA), the primary or only clinical manifestation of chronic cough, is a particular type of asthma [1]. As with asthma, the pathogenesis of CVA is not fully clear, and its pathophysiological changes are characterized by airway hyperresponsiveness and chronic inflammation [2]. CVA is a key pathogenic pathway for chronic cough and has been the most prevalent cause in China and Japan and the second in Korea, respectively [3–5]. Currently, the guidelines have suggested that regular inhaled corticosteroids and long-acting *β*2-agonists can be used as the recommended treatment modalities for patients with CVA [6, 7]. However, the long-term treatment effects of some CVA patients are not ideal, the recurrence rate is still high, and adverse reactions such as allergic reactions, as well as mental or neurological reactions, may occur [8, 9].

The main syndrome types of CVA in traditional Chinese medicine (TCM) theory are characterized by Qi deficiency and vigorous wind, which are generally classified as wind cough, stubborn cough, or wheezing cough [10]. Nowadays, combined with the TCM theory, TCM prescription has achieved a certain curative effect in the treatment of CVA [11, 12].

Huangqi Xixin decoction (HQXXD) is modified from the TCM prescriptions, Zhi Sou powders, and Yu Ping Feng powders [13]. It primarily comprises Huang Qi, Xi Xin, Jing Jie, Fang Feng, Huang Qin, Bai Zhu, Fu Ling, Chan Tui, Ban Xia, Bai Bu, and Gan Cao, whose pharmaceutical Latin names are Radix *Astragali* (RA), Herba cum Radix *Asari* (HRA), Herba *Schizonepetae* (HS), Radix *Saposhnikoviae* (RS), Radix *Scutellariae*, Rhizoma *Atractylodis macrocephalae*, Poria, *Periostracum cicadae*, Rhizoma *Pinelliae*, Radix *Stemonae,* and Radix *Glycyrrhizae*, respectively. RA, HRA, HS, and RS are the main active herbs in HQXXD, which are considered monarch and minister herbs according to the TCM theory. RA could tonify lung Qi; HRA, HS, and RS could tonify the spleen and stomach and dispel wind dampness to strengthen the effect of RA [14].

An earlier systematic review reported that there was no significant difference between the control and HQXXD groups in terms of the clinical efficacy reported by the total effective rate [13]. Our latest study, which included a meta-analysis of HQXXD's clinical curative effect on CVA, revealed that HQXXD acted on CVA in a variety of mechanisms, including through several compounds, targets, and pathways [14]. To date, numerous clinical studies using HQXXD for CVA have been published [15, 16]. However, the indicators evaluated were not comprehensive, such as recurrence rate, pulmonary function indices, and biochemical test indices. Therefore, a comprehensive and systematic review is needed to analyze the safety and effectiveness of HQXXD for CVA. This study aimed to comprehensively assess the safety and effectiveness of HQXXD for CVA.

## 2. Materials and Methods

### 2.1. Protocol and Registration

The protocol of the review was registered on the PROSPERO platform (https://www.crd.york.ac.uk/PROSPERO/) with the registration number CRD42021235772. PROSPERO, produced by the Centre for Reviews and Dissemination (CRD) and funded by the National Institute for Health Research (NIHR), is a global database of prospectively registered systematic reviews. At the beginning of the registration, PROSPERO can compare the completed review with the content of the protocol by offering a comprehensive listing of systematic reviews, to avoid the circumstances of duplication and reporting bias [17, 18]. Meanwhile, the review protocol has been published in an open-access journal [19].

The Preferred Reporting Items for Systematic Reviews and Meta-Analyses (PRISMA) 2020 statement served as the basis for our review [20, 21]. The PRISMA 2020 checklist is shown in Supplementary File 1.

### 2.2. Ethical Consideration

This systematic review collected data from open databases. All the eligible studies were approved by the local institutional ethics committee, and the written informed consent of the participants was collected. This systematic review did not directly involve the patient's privacy, so additional ethical approval was not necessary.

### 2.3. Search Strategy

We performed an overall search for published studies in main English and Chinese electronic databases from their inception to June 30, 2022, which include MEDLINE via PubMed, EMBASE via Ovid, Cochrane Central Register of Controlled Trials (CENTRAL), Chinese National Knowledge Infrastructure (CNKI), Chongqing VIP information (CQVIP), Wanfang database, and Chinese Biomedical Database (CBM). We tried to contact the authors to obtain the data we needed. The references in the included literature and systematic reviews were also inspected. On the basis of the databases feature, we modulated the search strategies of title, abstract, or keywords. Search strategies in PubMed are presented in Supplementary File 2, and the keywords “cough variant asthma” combined with “Huangqi Xixin decoction”; “Radix *Astragali*”; “Herba cum Radix *Asari*”; “Herba *Schizonepetae*”; “Radix *Saposhnikoviae*”; “traditional Chinese Medicine”; “Chinese Medicine”; and “herbal medicine” were used for the search. Some keywords were adjusted slightly in searching on different electronic databases.

### 2.4. Eligibility Criteria

The eligibility criteria strictly complied with PICOS (participant, intervention, comparison, outcome, and study design) principles.

#### 2.4.1. Study Design

Randomized controlled trials (RCTs) examining the safety and effectiveness of HQXXD for CVA were included in this review. We excluded case reports, retrospective observational studies, and animal research.

#### 2.4.2. Participants

Regardless of gender, age, race, educational level, and economic and marital status, individuals diagnosed with CVA using clearly defined or internationally recognized criteria were included.

Individuals with other respiratory conditions (bronchiectasis, chronic obstructive pulmonary disease), or severe liver, kidney, or heart disease were excluded.

#### 2.4.3. Intervention

Patients treated with HQXXD were included. HQXXD prescription can be modified, but it must contain monarch and minister herbs (RA, HRA, HS, and RS). Eligible treatments could be employed as monotherapy or combined conventional Western medicine treatment (CWMT).

#### 2.4.4. Comparison

Comparators included placebo, CWMT, or no interventions. Studies comparing other TCM prescription treatments in comparison were excluded.

#### 2.4.5. Outcomes

All the outcome indicators from the included studies were retrieved and evaluated. We performed a meta-analysis when the indicators could be subjected to a meta-analysis and a descriptive analysis when meta-analysis could not be done. The main outcomes included clinical efficacy, airway reactivity, recurrence rate, pulmonary function indices, biochemical test indices, and adverse events.

### 2.5. Study Selection, Data Extraction, and Quality Assessment

#### 2.5.1. Study Selection

All the literature was retrieved independently by three reviewers (Wang, Xia, and Hu). The initially identified references from searching databases were exported to NoteExpress 3.2.0 software. The duplicate studies were eliminated using NoteExpress software duplication models [22]. After that, the three reviewers independently evaluated each study's titles, abstracts, and keywords to screen possible eligible studies according to the predefined evaluation criteria. Finally, the three reviewers scrutinized and cross-checked the full text of previous eligible studies in the second stage and confirmed the final included studies for this systematic review. The reasons for excluding each study were recorded in detail in the second stage. A discussion was done to resolve the disagreements of the three reviewers at any stage of the selection process. Another reviewer (Zhang) was invited as an arbitrator to make a final decision when the disagreements could not be resolved after the discussion among the three reviewers. A PRISMA-compliant flow chart was used to outline the identification and selection procedures for this systematic review.

#### 2.5.2. Data Extraction and Dealing with Missing Data

Three reviewers (Wang, Xia, and Hu) conducted the data extraction procedure independently after completing a standard data extraction sheet. They cross-checked these results and examined whether there were any differences. A discussion was done to resolve the inconsistent opinions of the three reviewers. Another reviewer (Zhang) was invited as an arbitrator to judge these disagreements. The following list of information extracted from the original articles was saved in a standard data extraction sheet: (1) title, study year, country, and authors; (2) study design; (3) methodology: randomization, allocation concealment, patient and assessor blinding, inadequate outcome data, selective outcome reporting, and other risks of bias; (4) sample size; (5) age and gender in each group; (6) diagnostic criteria; (7) intervention group (methods of treatment, compositions of HQXXD prescription, duration of treatment); (8) comparison group (methods of treatment, duration of treatment); (9) main outcomes; and (11) adverse events. We contacted the corresponding authors of each study through email when the above information was missing.

#### 2.5.3. Quality Assessment

The quality of each eligible study was independently evaluated by three reviewers (Wang, Xia, and Hu) employing the Cochrane risk-of-bias tool from the Cochrane Handbook V.5.1.0. In addition to patient and assessor blinding, random sequence generation, allocation concealment, inadequate outcome data, selective outcome reporting, and other bias risks were among the seven criteria. Each item was categorized as having a low, high, or unclear risk of bias [23, 24]. In terms of other biases, we also carefully assessed the baseline imbalance and different sources of funding support. Disagreements were discussed with another reviewer (Zhang).

### 2.6. Statistical Analysis and Assessment of Heterogeneity

We used Review Manager (RevMan) 5.4 software to perform statistical analysis. To conduct a meta-analysis, we calculated the odds ratio (OR) with a 95% confidence interval (CI) for dichotomous data and the mean difference (MD) with a 95% CI for continuous data.

The chi-square and I^2^ tests were performed to explore the heterogeneity. When I^2^ < 50% and *P* > 0.1, there was homogeneity between each study. For the meta-analysis, the Mantel–Haenszel (M-H) fixed-effects model was employed. Otherwise, when I^2^ ≥ 50% or *P* < 0.1, the included studies were considered heterogeneous. Initially, to explore possible factors affecting the clinical heterogeneity, we re-assessed the demographic characteristics of the CVA patients and the variation of interventions between each included study. When clinical heterogeneity existed in this systematic review, we performed descriptive analysis. Otherwise, a random-effects model based on an inverse variance statistical approach was employed for further meta-analysis after getting rid of the clinical heterogeneity [25, 26]. When we performed the meta-analysis, *P* < 0.05 was taken as a significant value.

### 2.7. Subgroup Analysis

When heterogeneity was high and the number of included studies was sufficient, we assessed different interventions (monotherapy or combined CWMT), different control (placebo, CWMT, or no interventions), and different durations of treatment to carry out subgroup analysis [27].

### 2.8. Sensitivity Analysis

Based on methodological quality (low-quality studies were omitted to re-evaluate the results of this meta-analysis), statistical model (random-effects or fixed-effects model was used for analysis), and sample size (studies with smaller sample size were omitted to re-evaluate the outcomes of this meta-analysis), sensitivity analysis was carried out [28].

### 2.9. Publication Bias

When there were over five studies included in the meta-analysis, we used RevMan 5.4 software to assess publication bias with a funnel plot [29].

### 2.10. Summary of Evidence

We used the GRADEpro Web tool (https://gradepro.org/) to evaluate the quality of each main outcome in this systematic review and categorized them into 4 grades: high, medium, low, or very low. GRADEpro conducts the guideline development progression sternly depending on the GRADE methodology, including multiple fields of evidence such as summarization, recommendations, and dissemination. The judgments were based on the risk of bias, inconsistency, imprecision, indirectness, large effect, dose-response gradient, publication bias, and plausible confounding [30].

## 3. Results

### 3.1. Study Selection

**Figure 1:**
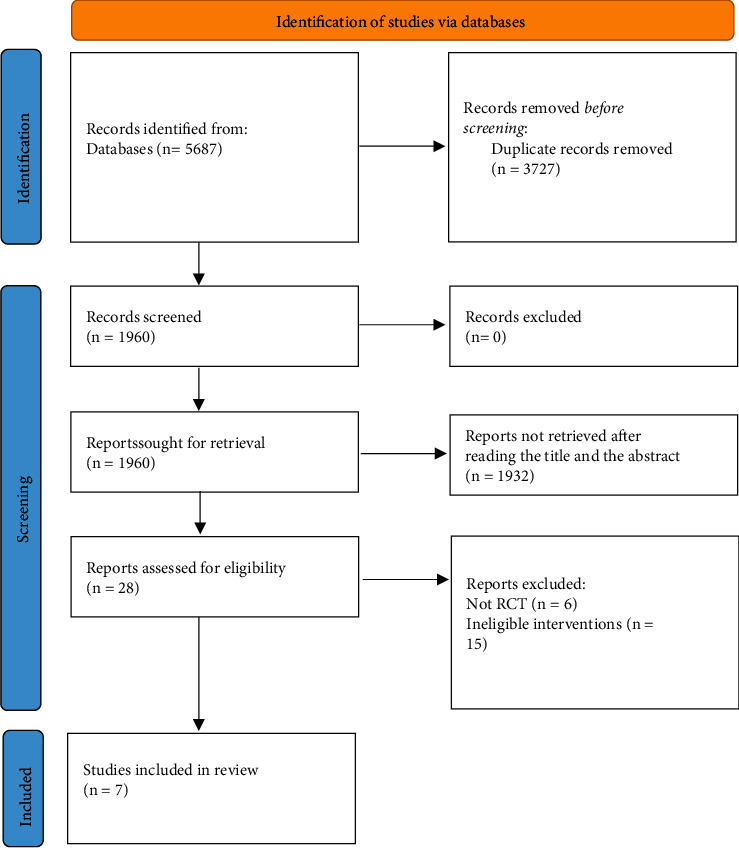
Flow diagram for the research selection process. Through database search, 5687 studies in total were retrieved. Finally, the systematic evaluation involved seven RCTs.

After excluding 3727 studies from the retrieved 5687 studies via database searching, because of the faint relevance expressed from the title and the abstract, 1932 were also removed from the remnant studies, and therefore, 28 studies were retained. Following the exclusion process of 21 studies due to various reasons, the systematic review eventually included seven RCTs [15, 16, 31–35]. In Figure 1, the findings of the literature screening and the process are displayed.

### 3.2. Description of Included Studies

Seven eligible RCTs were screened. All seven RCTs involving 512 patients were carried out in China. All of them were single-center studies. Participants in one study [16] were children, and participants in the other studies were adults. All studies used the prescription of HQXXD as monotherapy. The control group included CWMT; one study [15] used terbutaline sulfate tablets; one study [34] used procaterol hydrochloride tablets; three studies [31–33] used procaterol hydrochloride tablets and salbutamol aerosol; one study [35] used ambroxol hydrochloride tablets and terbutaline sulfate tablets; and one study [16] used montelukast sodium chewable tablets and budesonide aerosol.  Table 1 lists the basic features of the studies that were included, and Table 2 lists the compositions of HQXXD prescriptions used in each study's experimental group.

### 3.3. Methodological Quality

**Figure 2:**
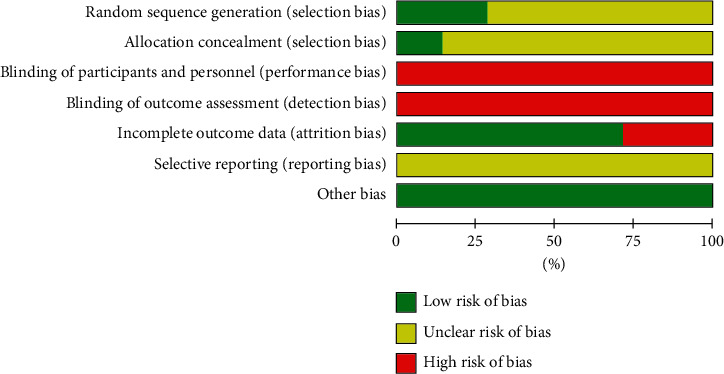
Risk-of-bias graph. Judgments about each risk-of-bias item as percentages across all included studies.

**Figure 3:**
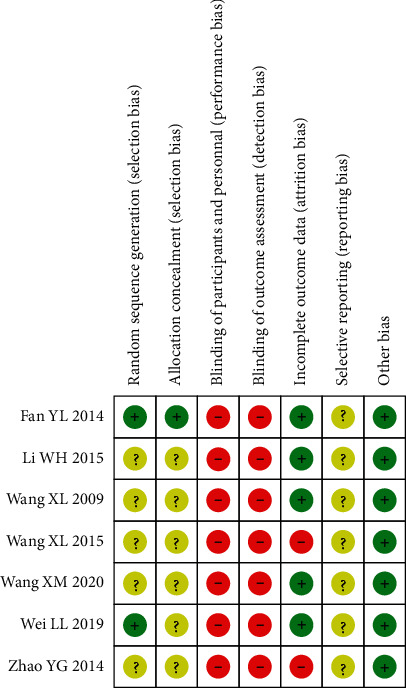
Risk-of-bias summary. Judgments about each risk-of-bias item for each included study. Green represents a low risk of bias, red represents a high risk of bias, and yellow represents an unclear risk of bias.

Two RCTs utilized adequate methods of random sequence generation, one [32] introduced the envelope method and another [35] introduced the draw random method, and other studies did not specifically describe the randomized methods and allocation concealment; none RCT introduced blindness; two RCT [31, 34] had incomplete outcome data; and selective reports cannot be identified from all studies. The risk-of-bias graph about each risk-of-bias item presented as percentages across all included studies is shown in Figure 2. In addition, the risk-of-bias summary about each risk-of-bias item for each included study is shown in Figure 3 and Table S1.

### 3.4. Outcomes

Six RCTs [15, 16, 32–35] compared the clinical efficacy reported by total effective rate; three RCTs [31, 32, 35] compared the total effective rate of airway responsiveness, and three RCTs [32–34] compared the recurrence rate, but one RCT [34] had incomplete data; two RCTs [31, 32] compared the improvement rate of cough, throat itching, and cough up phlegm, but one RCT [31] had incomplete data; one RCT [16] compared the serum tumor necrosis factor *α* (TNF-*α*), interleukin-8 (IL-8), and IL-6 indices, and pulmonary function indices including forced expiratory flow at 50% of forced vital capacity (FEF50), forced expiratory flow at 75% of forced vital capacity (FEF75), and maximal mid-expiratory flow (MMEF75/25). Adverse reactions were involved in the five studies [15, 32–35] with no adverse reactions reported, whereas the other two studies [16, 31] did not probe into adverse reactions.  Table 3 provides a summary of the key results.

### 3.5. Meta-Analysis

#### 3.5.1. Subgroup Analysis and Sensitivity Analysis

We first assessed the heterogeneity based on interventions, controls, and duration of treatment. When the heterogeneity was low, then subgroup analysis was not carried out. Sensitivity analysis was performed based on methodological quality, statistical model, and sample size. The sensitivity analysis demonstrated that the robustness and reliability of the pooled results were fair.

#### 3.5.2. Total Effective Rate of Clinical Efficacy

**Figure 4:**
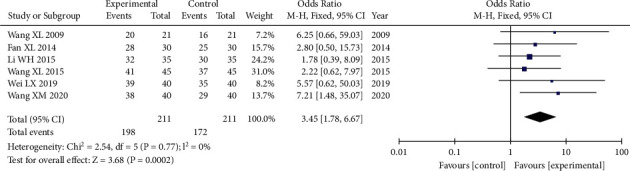
Forest plot of comparison: total effective rate of clinical efficacy. The pooled OR was 3.45 (95% CI [1.78–6.67], *P* < 0.0002). This showed that the total effective rate of clinical efficacy in the experimental group was substantially higher than that in the control group.

A total of 422 patients were included in the six studies [15, 16, 32–35] that compared the total effective rate of clinical efficacy with 211 in the experimental group and 211 in the control group. The heterozygosity test revealed that there was no heterogeneity in the six studies (*P*=0.77, I^2^ = 0%). When OR values were combined using the fixed-effects model, the pooled OR was 3.45 (95% CI [1.78–6.67], *P* < 0.0002). These findings demonstrated that *t* total effective rate of clinical efficacy in the experimental group was substantially higher than that of the control group (Figure 4).

#### 3.5.3. Total Effective Rate of Airway Responsiveness

**Figure 5:**
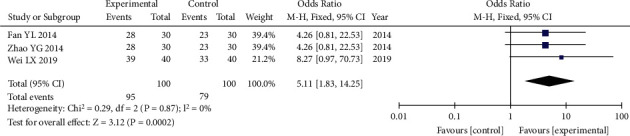
Forest plot of comparison: total effective rate of airway responsiveness. The pooled OR was 5.11 (95% CI [1.83–14.25], *P*=0.002). This showed that the experimental group-related total effective rate of airway responsiveness was considerably higher than that in the control group.

There were 200 individuals in total in the three studies [31, 32, 35] that compared the total effective rate of airway responsiveness, with 100 in the experimental group and 100 in the control group, respectively. The heterozygosity test revealed that there was no heterogeneity between the two studies (*P*=0.87, I^2^ = 0%). When OR values were combined using the fixed-effects model, the pooled OR was 5.11 (95% CI [1.83–14.25], *P*=0.002). This showed that the total effective rate of airway responsiveness in the experimental group was considerably higher than that in the control group (Figure 5).

#### 3.5.4. Recurrence Rate

**Figure 6:**
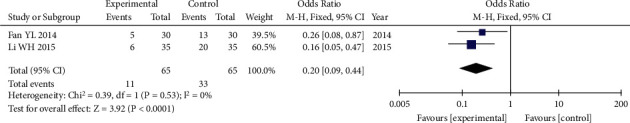
Forest plot of comparison: recurrence rate. The pooled OR was 0.20 (95% CI [0.09–0.44], *P* < 0.0001). This showed that the recurrence rate in the experimental group was considerably lower than that in the control group.

A total of 130 patients were included in the two studies [3, 33] that compared the recurrence rate, 65 of whom were in the experimental group and 65 in the control group, respectively. The heterozygosity test revealed that there was no heterogeneity between the two studies (*P*=0.53, I^2^ = 0%). The pooled OR was 0.20 (95% CI [0.09–0.44], *P* < 0.0001) when the fixed-effects model was applied to combine OR values. This showed that the recurrence rate in the experimental group was considerably lower than that in the control group (Figure 6).

#### 3.5.5. Publication Bias Analysis

**Figure 7:**
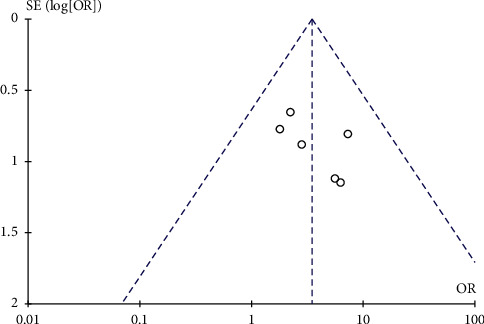
Funnel plots of clinical efficacy presented by total effective rate. Each outcome's OR value is displayed by the horizontal coordinate, while the longitudinal coordinates indicate SE (log [OR]). The funnel plots revealed a basically symmetrical and inverted funnel form. The outcomes demonstrated that there is not any obvious publication bias.

Utilizing funnel plots, the publication bias was investigated. We created funnel plots showing the clinical efficacy as presented as the total effective rate, where the horizontal coordinate was each outcome OR value and the longitudinal coordinate was the SE (log [OR]). The funnel plots are shown in Figure 7.

### 3.6. Safety of HQXXD for CVA

Five studies [15, 32–35] involved adverse reactions and reported that HQXXD had no adverse reaction in treatment. Other studies [16, 31] did not probe into the adverse reactions. HQXXD is safe for CVA patients.

### 3.7. GRADE Level of Evidence

**Figure 8:**
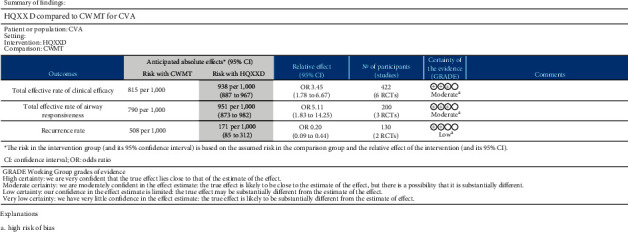
GRADE levels of HQXXD compared with CWMT for CVA evidence.

The GRADE levels of evidence were moderate for total effective rates of clinical efficacy and airway responsiveness outcomes. For the outcome of recurrence rate, the GRADE level of evidence was low. The GRADE evidence profiles are shown in Figure 8. The high risk of bias was the primary cause of a decreasing level.

## 4. Discussion

Based on tonifying lung Qi and dispelling wind dampness, TCM has been used in the treatment of CVA and the treatment effect is clear [12, 36]. HQXXD, in which RA, RS, HRA, and HS are monarch and minister herbs, could tonify lung Qi and dispel wind dampness to treat CVA according to the TCM theory [13]. On the basis of a network pharmacology approach, a previous study interrogated the multicomponent, multitarget, and multi-pathway characteristics of HQXXD acting on CVA [14]. Basic and clinical study of HQXXD treating CVA deserves further research.

However, there is no systematic review showing comprehensive evidence regarding the safety and effectiveness of HQXXD in CVA. This systematic review illustrated that total effective rates of clinical efficacy and airway responsiveness were considerably higher in the HQXXD group in comparison with those in the CWMT group (both had *P* < 0.05); the recurrence rate was considerably lower in the HQXXD group in comparison with that in CWMT group (*P* < 0.05).

The total effective rate of clinical efficacy is the most intuitive index of clinical efficacy evaluation. When comparing the total effective rate of clinical efficacy between the HQXXD and the CWMT groups, individual studies [15, 32–35] did not find a considerable difference. In the previous meta-analyses, one study [13] showed no significant difference, whereas one study [14] showed a statistically significant difference. Based on expanding the sample size, this systematic review confirmed that the total effective rate of clinical efficacy in the HQXXD group was substantially higher than that of the CWMT group.

Airway responsiveness is an important pathophysiological mechanism in CVA [37]. When comparing the total effective rate of airway responsiveness between the HQXXD and the CWMT groups, individual studies [31, 32, 35] did not find a statistically significant difference. This systematic review clarified that the HQXXD group had a substantially higher total effective rate of airway responsiveness than the CWMT group.

Through CWMT, the long-term treatment effects of some CVA patients are not ideal and the recurrence rate is still high [8, 9]. There is no systematic review regarding the recurrence rate of HQXXD for CVA patients. This systematic review was the first to report that the recurrence rate in the HQXXD group was substantially lower than that in the CWMT group.

Regarding the safety of HQXXD for CVA, five studies [15, 32–35] regarding adverse reactions showed that HQXXD had no adverse reaction in treatment. Other studies [16, 31] did not involve adverse reactions. We did a descriptive analysis that HQXXD is safe for CVA patients.

Moreover, two RCTs [31, 32] compared the improvement rate of cough, throat itching, and cough up phlegm; one RCT [16] compared serum TNF-*α*, IL-8, and IL-6 indices, and FEF50, FEF75, and MMEF75/25. These provide references for our in-depth research and need more relevant studies to conduct a further evaluation.

This systematic review is aimed at highlighting the HQXXD prescription for routine CNA treatment, or providing a basis for further real-world RCT research of HQXXD and obtaining more evidence-based medical evidence for better application of TCM. Our review protocol was registered on the PROSPERO platform, which is produced by CRD and funded by the NIHR, aiming to avoid the circumstances of duplication and reporting bias. On the other hand, this review was performed based on the PRISMA, and all of these make our review more standardized and reliable.

One of the seven studies [16] selected for final analysis includes only children. Our inclusion and exclusion criteria did not limit the age, so it was eventually included. We performed heterogeneity analysis, which showed no obvious heterogeneity, and the number of included studies was less, so we did not conduct subgroup analysis. With the publication of more relevant RCT, further subgroup analysis may be possible to obtain more accurate evidence.

However, this systematic review may have some potential limitations. First of all, HQXXD prescription originates in China, and the results of this review may limit to Asian patients although the internationalization of TCM is becoming increasingly extensive. Secondly, some of the studies do not introduce allocation concealment and contain unclear random methods; the studies lack the introduction of blindness and the ability to ensure the presence of selective reports; two studies had incomplete outcome data. Despite the low-quality research methods, the careful evaluation of literature can be used to compensate for the facticity and creditability of results. Lastly, besides the different dosages of HQXXD compositions and the drugs in the control group, the methods, as well as duration of treatment, were not uniform. The presence of these biases might skew the research's results. However, our research primarily focused on the use of HQXXD, particularly RA, HRA, HS, and RS for individuals with CVA, so there is no special regulation on the dose. The baselines for inclusion in the literature are not considerably different, and the studies that were included were RCTs with consistent diagnostic criteria. Based on interventions, controls, and duration of treatment, we assessed the heterogeneity as low. Based on methodological quality, statistical model, and sample size, the sensitivity analysis demonstrated that the robustness and reliability of the pooled results were fair. It was evident from the funnel plot that there is no remarkable publishing bias. Furthermore, for total effective rates of clinical efficacy and airway responsiveness outcomes, the GRADE levels of evidence were moderate; for recurrence rate outcomes, the GRADE level of evidence is low. The high risk of bias was the main reason for a lower level; high-quality and multicenter RCTs are required to produce better evidence.

## 5. Conclusion

In brief, HQXXD is safe and effective in the treatment of CVA patients, which benefits clinical efficacy and airway responsiveness, reduces the recurrence rate, and has no adverse effects. More high-quality, multicenter, large-sample RCTs are required for the purpose of gathering better evidence because of the potential risk of bias. This systematic review offers more evidence regarding the effectiveness and safety of HQXXD for CVA and helps clinicians in decision-making when treating CVA patients. In addition, it can provide a basis for future basic and clinical research.

## Figures and Tables

**Table 1:** Summary of RCTs of HQXXD for CVA.

Study year [ref]	Country	Sample size (experimental/control)	Mean age (years) (experimental/control)	Experimental	Control	Duration
Wang and Xie 2009 [15]	China	42 (21/21)	34.5 ± 2.7/33.2 ± 2.6	HQXXD	Terbutaline sulfate tablets	3 weeks
Zhao 2014 [31]	China	90 (45/45)	42.1 ± 8.27/39.1 ± 11.30	HQXXD	Procaterol hydrochloride tablets + salbutamol aerosol	4 weeks
Fan and Xie 2014 [32]	China	60 (30/30)	42.10 ± 8.27/38.2 ± 10.35	HQXXD	Procaterol hydrochloride tablets + salbutamol aerosol	4 weeks
Li 2015 [33]	China	70 (35/35)	50 ± 1.5/49 ± 1.3	HQXXD	Procaterol hydrochloride tablets + salbutamol aerosol	4 weeks
Wang and Xie 2015 [34]	China	90 (45/45)	41.12 ± 7.24/40.18 ± 9.35	HQXXD	Procaterol hydrochloride tablets	4 weeks
Wei and Qin 2019 [35]	China	80 (40/40)	45.35 ± 3.88/43.25 ± 3.78	HQXXD	Ambroxol hydrochloride tablets + terbutaline sulfate tablets	4 weeks
Wang 2020 [16]	China	80 (40/40)	4.56 ± 2.28/4.62 ± 2.13	HQXXD	Montelukast sodium chewable tablets + budesonide aerosol	8 weeks

RCT: randomized controlled trial, HQXXD: Huangqi Xixin decoction, and CVA: cough variant asthma.

**Table 2:** Compositions of TCM prescriptions.

Study year (ref)	TCM prescriptions	Compositions of TCM prescriptions		
		Latin name	English name	Chinese name
Wang and Xie 2009 [15]	HQXXD	Radix *Astragali*	*Astragalus* root	Huang Qi
		Herba cum Radix *Asari*	Asarum	Xi Xin
		Herba *Schizonepetae*	Schizonepeta stem or bud	Jing Jie
		Radix *Saposhnikoviae*	Saposhnikoviae root	Fang Feng
		Radix *Scutellariae*	Scute	Huang Qin
		*Ramulus Cinnamomi*	Cinnamon twig	Gui Zhi
		Rhizoma *Atractylodis macrocephalae*	Atractylodis rhizome	Bai Zhu
		Poria	Tuckahoe	Fu Ling
		*Periostracum cicadae*	Cicada molting (slough)	Chan Tui
		Rhizoma *Pinelliae*	Pinellia rhizome	Ban Xia
		Radix *Stemonae*	Stemona root	Bai Bu
		Radix *Glycyrrhizae*	Licorice root	Gan Cao
		Rhizoma *Zingiberis Recens*	Fresh ginger rhizome	Sheng Jiang
Zhao 2014 [31]	HQXXD	Radix *Astragali*	*Astragalus* root	Huang Qi
		Herba cum Radix *Asari*	Asarum	Xi Xin
		Herba *Schizonepetae*	Schizonepeta stem or bud	Jing Jie
		Radix *Saposhnikoviae*	Saposhnikoviae root	Fang Feng
		Radix *Scutellariae*	Scute	Huang Qin
		Rhizoma *Atractylodis macrocephalae*	Atractylodis rhizome	Bai Zhu
		Poria	Tuckahoe	Fu Ling
		*Periostracum cicadae*	Cicada molting (slough)	Chan Tui
		Rhizoma *Pinelliae*	Pinellia rhizome	Ban Xia
		Radix *Stemonae*	Stemona root	Bai Bu
		Radix *Glycyrrhizae*	Licorice root	Gan Cao
		Rhizoma *Zingiberis Recens*	Fresh ginger rhizome	Sheng Jiang
Fan and Xie 2014 [32]	HQXXD	Radix *Astragali*	*Astragalus* root	Huang Qi
		Herba cum Radix *Asari*	Asarum	Xi Xin
		Herba *Schizonepetae*	Schizonepeta stem or bud	Jing Jie
		Radix *Saposhnikoviae*	Saposhnikoviae root	Fang Feng
		Radix *Scutellariae*	Scute	Huang Qin
		Rhizoma *Atractylodis macrocephalae*	Atractylodis rhizome	Bai Zhu
		Poria	Tuckahoe	Fu Ling
		*Periostracum cicadae*	Cicada molting (slough)	Chan Tui
		Rhizoma *Pinelliae*	Pinellia rhizome	Ban Xia
		Radix *Stemonae*	Stemona root	Bai Bu
		Radix *Glycyrrhizae*	Licorice root	Gan Cao
		Rhizoma *Zingiberis Recens*	Fresh ginger rhizome	Sheng Jiang
Li 2015 [33]	HQXXD	Radix *Astragali*	*Astragalus* root	Huang Qi
		Herba cum Radix *Asari*	Asarum	Xi Xin
		Herba *Schizonepetae*	Schizonepeta stem or bud	Jing Jie
		Radix *Saposhnikoviae*	Saposhnikoviae root	Fang Feng
		Radix *Scutellariae*	Scute	Huang Qin
		Rhizoma *Atractylodis macrocephalae*	Atractylodis rhizome	Bai Zhu
		Poria	Tuckahoe	Fu Ling
		*Periostracum cicadae*	Cicada molting (slough)	Chan Tui
		Rhizoma *Pinelliae*	Pinellia rhizome	Ban Xia
		Radix *Stemonae*	Stemona root	Bai Bu
		Radix *Glycyrrhizae*	Licorice root	Gan Cao
		Rhizoma *Zingiberis Recens*	Fresh ginger rhizome	Sheng Jiang
Wang and Xie 2015 [34]	HQXXD	Radix *Astragali*	*Astragalus* root	Huang Qi
		Herba cum Radix *Asari*	Asarum	Xi Xin
		Herba *Schizonepetae*	Schizonepeta stem or bud	Jing Jie
		Radix *Saposhnikoviae*	Saposhnikoviae root	Fang Feng
		Radix *Scutellariae*	Scute	Huang Qin
		Rhizoma *Atractylodis macrocephalae*	Atractylodis rhizome	Bai Zhu
		Poria	Tuckahoe	Fu Ling
		*Periostracum cicadae*	Cicada molting (slough)	Chan Tui
		Rhizoma *Pinelliae*	Pinellia rhizome	Ban Xia
		Radix *Stemonae*	Stemona root	Bai Bu
		Radix *Glycyrrhizae*	Licorice root	Gan Cao
		Rhizoma *Zingiberis Recens*	Fresh ginger rhizome	Sheng Jiang
Wei and Qin 2019 [35]	HQXXD	Radix *Astragali*	*Astragalus* root	Huang Qi
		Herba cum Radix *Asari*	Asarum	Xi Xin
		Herba *Schizonepetae*	Schizonepeta stem or bud	Jing Jie
		Radix *Saposhnikoviae*	Saposhnikoviae root	Fang Feng
		Radix *Scutellariae*	Scute	Huang Qin
		Rhizoma *Atractylodis macrocephalae*	Atractylodis rhizome	Bai Zhu
		Poria	Tuckahoe	Fu Ling
		*Periostracum cicadae*	Cicada molting (slough)	Chan Tui
		Rhizoma *Pinelliae*	Pinellia rhizome	Ban Xia
		Radix *Stemonae*	Stemona root	Bai Bu
		Radix *Glycyrrhizae*	Licorice root	Gan Cao
		Rhizoma *Zingiberis Recens*	Fresh ginger rhizome	Sheng Jiang
Wang 2020 [16]	HQXXD	Radix *Astragali*	*Astragalus* root	Huang Qi
		Herba cum Radix *Asari*	Asarum	Xi Xin
		Herba *Schizonepetae*	Schizonepeta stem or bud	Jing Jie
		Radix *Saposhnikoviae*	Saposhnikoviae root	Fang Feng
		Radix *Scutellariae*	Scute	Huang Qin
		Rhizoma *Atractylodis macrocephalae*	Atractylodis rhizome	Bai Zhu
		Poria	Tuckahoe	Fu Ling
		*Periostracum cicadae*	Cicada molting (slough)	Chan Tui
		Rhizoma *Pinelliae*	Pinellia rhizome	Ban Xia
		Radix *Stemonae*	Stemona root	Bai Bu
		Radix *Glycyrrhizae*	Licorice root	Gan Cao
		Rhizoma *Zingiberis Recens*	Fresh ginger rhizome	Sheng Jiang

TCM: traditional Chinese medicine; HQXXD: Huangqi Xixin decoction.

**Table 3:** Main outcomes of included RCTs.

Study year (ref)	Main outcomes	Main results (effect size)	Adverse events
Wang and Xie 2009 [15]	Total effective rate of clinical efficacy	OR 6.25 [0.66, 59.03]	No adverse reaction
Zhao 2014 [31]	Improvement rate of cough	*P* < 0.05 (no specific data)	Not reported
	Improvement rate of throat itching	*P* < 0.05 (no specific data)	
	Improvement rate of cough up phlegm	*P* < 0.05 (no specific data)	
	Total effective rate of airway responsiveness	OR 4.26 [0.81, 22.53]	
Fan and Xie 2014 [32]	Total effective rate of clinical efficacy	OR 2.80 [0.50, 15.73]	No adverse reaction
	Improvement rate of cough	OR 1.56 [0.53, 4.53]	
	Improvement rate of throat itching	OR 1.41 [0.45, 4.45]	
	Improvement rate of cough up phlegm	OR 1.59 [0.53, 4.77]	
	Total effective rate of airway responsiveness	OR 4.26 [0.81, 22.53]	
	Recurrence rate	OR 0.26 [0.08, 0.87]	
Li 2015 [33]	Total effective rate of clinical efficacy	OR 1.78 [0.39, 8.09]	No adverse reaction
	Recurrence rate	0.16 [0.05, 0.47]	
Wang and Xie 2015 [34]	Total effective rate of clinical efficacy	OR 2.22 [0.62, 7.97]	No adverse reaction
	Recurrence rate	*P* < 0.05 (no specific data)	
Wei and Qin 2019 [35]	Total effective rate of clinical efficacy	OR 5.57 [0.62, 50.03]	No adverse reaction
	Total effective rate of airway responsiveness	OR 8.27 [0.97, 70.73]	
Wang 2020 [16]	Total effective rate of clinical efficacy	OR 7.21 [1.48, 35.07]	Not reported
	Serum TNF-*α*	MD −24.12 [−27.72, −20.52]	
	Serum IL-8	MD −3.81 [−4.67, −2.95]	
	Serum IL-6	MD −6.53 [−7.06, −6.00]	
	FEF50	MD 13.83 [8.92, 18.74]	
	FEF75	MD 14.56 [10.35, 18.77]	
	MMEF75/25	MD 16.00 [11.61, 20.39]	

RCT: randomized controlled trial, TNF: tumor necrosis factor, IL: interleukin, FEF50: forced expiratory flow at 50% of forced vital capacity, FEF75: forced expiratory flow at 75% of forced vital capacity, MMEF75/25: maximal mid-expiratory flow, OR: odds ratio, and MD: mean difference.

## Data Availability

The extracted data used to support the findings of this study are available from the corresponding author upon request.

## Supplementary Materials

Supplementary Materials
*Supplementary File 1.* PRISMA 2020 checklist. *Supplementary File 2*. Search strategy in PubMed for example. *Table S1*. Risk of bias of included RCTs.

## References

[1] Zhang L., Liu S., Li M., Xu X. (2020). Diagnostic value of fractional exhaled nitric oxide in cough-variant asthma: an updated meta-analysis. *Journal of Asthma*.

[2] Gao J., Wu F., Wu S., Yang X. (2020). Inflammatory subtypes in classic asthma and cough variant asthma. *Journal of Inflammation Research*.

[3] Lai K., Long L. (2020). Current status and future directions of chronic cough in China. *Lung*.

[4] Niimi A. (2016). Cough variant asthma. *Nihon Rinsho*.

[5] An T. J., Kim J. W., Choi E. Y. (2020). Clinical characteristics of chronic cough in Korea. *Tuberculosis and Respiratory Diseases*.

[6] Dicpinigaitis P. V. (2006). Chronic cough due to asthma: ACCP evidence-based clinical practice guidelines. *Chest*.

[7] Morice A. H., Millqvist E., Bieksiene K. (2020). ERS guidelines on the diagnosis and treatment of chronic cough in adults and children. *European Respiratory Journal*.

[8] Bao W., Chen Q., Lin Y. (2013). Efficacy of procaterol combined with inhaled budesonide for treatment of cough-variant asthma. *Respirology*.

[9] Tagaya E., Kondo M., Kirishi S., Kawagoe M., Kubota N., Tamaoki J. (2015). Effects of regular treatment with combination of salmeterol/fluticasone propionate and salmeterol alone in cough variant asthma. *Journal of Asthma*.

[10] Chen D., Zhang F., Tang S. (2013). A network-based systematic study for the mechanism of the treatment of Zhengs related to cough variant asthma. *Evidence-Based Complementary and Alternative Medicine*.

[11] Miao Q., Wei P. C., Fan M. R., Zhang Y. P. (2013). Clinical study on treatment of cough variant asthma by Chinese medicine. *Chinese Journal of Integrative Medicine*.

[12] Gu C., Peng W., Wang Z., Xu Y., Han D., Zhou X. (2020). Suhuang zhike capsules for the treatment of cough variant asthma: a meta-analysis. *Evidence-Based Complementary and Alternative Medicine*.

[13] Chen L., Qi J., Zhang Y. F., Jiang W. L. (2016). Systematic review of Astragalus Asarum decoction in the treatment of cough variant asthma. *World Chinese Medicine*.

[14] Xia Q., Liu M., Li H., Tian L., Qi J., Zhang Y. (2020). Network pharmacology strategy to investigate the pharmacological mechanism of Huangqi Xixin decoction on cough variant asthma and evidence-based medicine approach validation. *Evidence-Based Complementary and Alternative Medicine*.

[15] Wang X. L., Xie M. H. (2009). Treatment of 21 cases of cough variant asthma with Astragalus Asarum decoction. *Journal of Emergency in Traditional Chinese Medicine*.

[16] Wang X. M. (2020). Clinical observation of Huangqi Xixin decoction in treating cough variant asthma. *Journal of Practical Traditional Chinese Medicine*.

[17] Stewart L., Moher D., Shekelle P. (2012). Why prospective registration of systematic reviews makes sense. *Systematic Reviews*.

[18] Moher D., Booth A., Stewart L. (2014). How to reduce unnecessary duplication: use PROSPERO. *BJOG: An International Journal of Obstetrics and Gynaecology*.

[19] Xia Q., Wang C., Zhang Y., Jiang W., Qi J. (2022). The effectiveness and safety of HuangQiXiXin decoction for cough variant asthma: protocol for a systematic review. *Clinical Research Communications*.

[20] Page M. J., McKenzie J. E., Bossuyt P. M. (2021). The PRISMA 2020 statement: an updated guideline for reporting systematic reviews. *British Medical Journal*.

[21] Page M. J., Moher D., Bossuyt P. M. (2021). PRISMA 2020 explanation and elaboration: updated guidance and exemplars for reporting systematic reviews. *British Medical Journal*.

[22] Zhang S., Chen Z. L., Tang Y. P., Duan J. L., Yao K. W. (2021). Efficacy and safety of Xue-Fu-Zhu-Yu decoction for patients with coronary heart disease: a systematic review and meta-analysis. *Evidence-Based Complementary and Alternative Medicine*.

[23] Higgins J. P. T., Altman D. G., Gotzsche P. C. (2011). The cochrane collaboration’s tool for assessing risk of bias in randomised trials. *British Medical Journal*.

[24] Zhang Y., Gu L., Xia Q., Tian L., Qi J., Cao M. (2020). Radix Astragali and Radix angelicae sinensis in the treatment of idiopathic pulmonary fibrosis: a systematic review and meta-analysis. *Frontiers in Pharmacology*.

[25] Higgins J. P. T., Thompson S. G. (2002). Quantifying heterogeneity in a meta-analysis. *Statistics in Medicine*.

[26] Higgins J. P. T., Thompson S. G., Deeks J. J., Altman D. G. (2003). Measuring inconsistency in meta-analyses. *British Medical Journal*.

[27] Low Z. X., Yeo K. A., Sharma V. K. (2019). Prevalence of burnout in medical and surgical residents: a meta-analysis. *International Journal of Environmental Research and Public Health*.

[28] Taeuber I., Weibel S., Herrmann E. (2021). Association of intravenous tranexamic acid with thromboembolic events and mortality: a systematic review, meta-analysis, and meta-regression. *Journal of the American Medical Association Surgery*.

[29] Fan Y., Zhang Q., Tian R., Jiang J. (2017). The effect of foot bath of Chinese medicine combined with acupoint injection for diabetic peripheral neuropathy: a meta analysis. *Traditional Medicine Research*.

[30] Guyatt G., Oxman A. D., Akl E. A. (2011). GRADE guidelines: 1. Introduction-GRADE evidence profiles and summary of findings tables. *Journal of Clinical Epidemiology*.

[31] Zhao Y. G. (2014). Clinical observation of *Astragalus asarum* decoction in treating cough variant asthma (Qi deficiency and wind vigorous). *Medical Aesthetics and Cosmetology*.

[32] Fan Y. L., Xie M. H. (2014). Clinical observation of Astragalus Asarum decoction in treating cough variant asthma (Qi deficiency and wind vigorous). *Journal of Emergency in Traditional Chinese Medicine*.

[33] Li W. H. (2015). Clinical observation of Huangqi Xixin decoction in treating cough variant asthma. *The Medical Forum*.

[34] Wang X. L., Xie M. H. (2015). Treatment of 45 cases of cough variant asthma with Yiqi Shufeng. *Henan Traditional Chinese Medicine*.

[35] Wei L. X., Qin Q. J. (2019). Clinical efficacy and safety evaluation of Huangqi Xixin decoction in treating cough variant asthma. *World Latest Medicine Information*.

[36] Kyou-Hwan H., Ki Haeng C., Cui S. Q., Lily L., Jaejong K. (2021). Effectiveness and safety of traditional Chinese herbs in children with cough variant asthma: a systematic review and meta-analysis. *Journal of Traditional Chinese Medicine*.

[37] Yi F., Han L., Liu B. (2020). Determinants of response to bronchodilator in patients with cough variant asthma- A randomized, single-blinded, placebo-controlled study. *Pulmonary Pharmacology & Therapeutics*.

