# Supplementary material for: The Effectiveness and Safety of Huangqi Xixin Decoction for Cough Variant Asthma: A Systematic Review and Meta-Analysis
Source: Evid Based Complement Alternat Med. 2022 Sep 20;2022:9492100. doi: 10.1155/2022/9492100 (PMC9526668; doi:10.1155/2022/9492100)
Supplement: Supplementary Materials — Supplementary File 1. PRISMA 2020 checklist. Supplementary File 2. Search strategy in PubMed for example. Table S1. Risk of bias of included RCTs. [file 9492100.f1.zip › Supplementary File 2 Search strategy in PubMed (1).pdf]

Supplementary File 2

Search strategy in PubMed for example

((((((((Huangqi Xixin decoction[Text Word]) OR (Radix Astragali[Text Word])) OR (Herba cum Radix Asari[Text Word])) OR (Herba Schizonepetae[Text Word])) OR (Radix Saposhnikoviae[Text Word])) OR (traditional Chinese Medicine[Text Word])) OR (Chinese Medicine[Text Word])) OR (herbal medicine[Text Word])) AND (cough variant asthma[Text Word])
