# Supplementary material for: The Effectiveness and Safety of Huangqi Xixin Decoction for Cough Variant Asthma: A Systematic Review and Meta-Analysis
Source: Evid Based Complement Alternat Med. 2022 Sep 20;2022:9492100. doi: 10.1155/2022/9492100 (PMC9526668; doi:10.1155/2022/9492100)
Supplement: Supplementary Materials — Supplementary File 1. PRISMA 2020 checklist. Supplementary File 2. Search strategy in PubMed for example. Table S1. Risk of bias of included RCTs. [file 9492100.f1.zip › Table S1 Risk of bias of included RCTs.pdf]

Table S1 Risk of bias of included RCTs

Study year[ref]	Random sequence generation	Allocation concealment	Blinding of patient	Blinding of assessor	Incomplete outcome data	Selective reporting	Other bias
Wang XL 2009[15]	U	U	H	H	L	U	L
Zhao YG 2014[31]	U	U	H	H	H	U	L
Fan YL 2014[32]	L	L	H	H	L	U	L
Li WH	U	U	H	H	L	U	L

2015[33]							
Wang XL 2015[34]	U	U	H	H	H	U	L
Wei LX 2019[35]	L	U	H	H	L	U	L
Wang XM 2020[16]	U	U	H	H	L	U	L

RCT: randomized controlled trial, L: low risk of bias, H: high risk of bias, U: unclear risk of bias.
